# Characterization of glomerular extracellular matrix in IgA nephropathy by proteomic analysis of laser-captured microdissected glomeruli

**DOI:** 10.1186/s12882-019-1598-1

**Published:** 2019-11-14

**Authors:** Flavia Teodora Ioana Paunas, Kenneth Finne, Sabine Leh, Tarig Al-Hadi Osman, Hans-Peter Marti, Frode Berven, Bjørn Egil Vikse

**Affiliations:** 1 Department of Medicine, Haugesund Hospital, Haugesund, Norway; 2 Department of Clinical Medicine, University of Bergen, Bergen, Norway; 3 Department of Pathology, Haukeland University Hospital, Bergen, Norway; 4 Department of Medicine, Haukeland University Hospital, Bergen, Norway; 5 Department of Biomedicine, University of Bergen, Bergen, Norway

**Keywords:** IgA nephropathy, Glomerulonephritis, Proteomics, ESRD

## Abstract

**Background:**

IgA nephropathy (IgAN) involves mesangial matrix expansion, but the proteomic composition of this matrix is unknown. The present study aimed to characterize changes in extracellular matrix in IgAN.

**Methods:**

In the present study we used mass spectrometry-based proteomics in order to quantitatively compare protein abundance between glomeruli of patients with IgAN (*n* = 25) and controls with normal biopsy findings (*n* = 15).

**Results:**

Using a previously published paper by Lennon et al. and cross-referencing with the Matrisome database we identified 179 extracellular matrix proteins. In the comparison between IgAN and controls, IgAN glomeruli showed significantly higher abundance of extracellular matrix structural proteins (e.g periostin, vitronectin, and extracellular matrix protein 1) and extracellular matrix associated proteins (e.g. azurocidin, myeloperoxidase, neutrophil elastase, matrix metalloproteinase-9 and matrix metalloproteinase 2). Periostin (fold change 3.3) and azurocidin (3.0) had the strongest fold change between IgAN and controls; periostin was also higher in IgAN patients who progressed to ESRD as compared to patients who did not.

**Conclusion:**

IgAN is associated with widespread changes of the glomerular extracellular matrix proteome. Proteins important in glomerular sclerosis or inflammation seem to be most strongly increased and periostin might be an important marker of glomerular damage in IgAN.

## Background

IgA nephropathy (IgAN) is the most prevalent primary chronic glomerular disease worldwide [1], and although benign in many cases, it is reported that up to 30–50% will slowly progress to end stage renal failure [2, 3]. The pathogenesis of IgAN involves mesangial deposition of immune-complexes containing galactose deficient IgA1 that leads to mesangial cell activation and initiation of glomerular injury [4]. Mesangial proliferation is scored in the Oxford classification of IgAN and has been shown to predict progressive disease [5]. Activated mesangial cells secrete inflammatory mediators and components of the extracellular matrix, and mesangial hypercellularity is thus morphologically often associated with increase of extracellular matrix (ECM).

The glomerular extracellular matrix is a dynamic structure which acts both as structural support for the cells and as an active component in cell signaling [6]. Lennon et al. previously described the protein composition of the glomerular ECM, identifying 144 structural and regulatory ECM proteins [7]. Recently, Hobeika et al. [8] expanded the list by including their identified proteins from microdissected glomerular tissue and comparing with proteins described in the Matrisome project database - a curated database of ECM structural and associated proteins [9], http://matrisomeproject.mit.edu/. IgAN is a disease characterized by glomerular ECM expansion, but the glomerular proteomic changes have not been investigated in detail.

In the present study we microdissected glomerular tissue from 25 patients with IgAN and 15 patients with normal findings on kidney biopsy (controls) in order to investigate glomerular ECM proteins which either had been defined by Lennon et al. [7] or by the Matrisome project database. Our hypothesis was that composition of glomerular ECM would be changed in IgAN as compared to controls and that some ECM proteins would also be associated with progressive clinical course in IgAN. An improved understanding of these changes may be important for a better understanding of the glomerular damage in IgAN.

## Methods

### Registries used in the study

The patients were selected from Norwegian Kidney Biopsy Registry. Data on end stage renal disease (ESRD) were retrieved by linking data with data from the Norwegian Renal Registry. We calculated GFR based on The chronic kidney disease - Epidemiology Collaboration (CKD-EPI) equation [10]. Urinary protein was quantified as grams/24 h, as previously described [11], either from directly measured values, by calculation from reported urinary protein to creatinine ratio or if only reported by urinary dipstick a negative dipstick was set to 0 g/24 h, 1+ was set to 0.5 g/24 h, 2+ was set to 1.0 g/24 h and 3+ was set to 3.0 g/24 h.

### Study population

IgAN patients with an estimated glomerular filtration rate (GFR) of > 45 ml/min/1.73m^2^ were selected for the study. In total 25 IgAN patients were included in the study, these were divided based on whether or not they progressed to ESRD (defined as time to dialysis or transplantation) during the first 10 years after being diagnosed: 16 patients with non-progressive IgAN and 9 patients with progressive IgAN. In addition, 15 patients with normal findings on kidney biopsy (most common biopsy indication hematuria and/or microalbuminuria) and with estimated glomerular filtration rate of > 60 ml/min/1.73m^2^, proteinuria < 0.5 g/24 h and available kidney tissue were included as controls (only one control patient had GFR < 90 ml/min/1.73m^2^). The study was approved by the Regional Committee for Medical and Health Research Ethics (approval number 2013/553).

### Laser capture microdissection

As previously described [11], formalin-fixed paraffin-embedded tissue (FFPE) from the remaining part of the kidney biopsy core that was not used for diagnostics was cut into five micrometer thick sections, mounted on pre-irradiated polyethylene naphthalate slides, deparaffinized and stained with hematoxylin eosin. Approximately 100 glomerular cross sections were laser microdissected by the first author and collected into specialized tubes for each sample. We excluded glomeruli with global sclerosis, more than minimal segmental sclerosis, crescents or fibrinoid necrosis. Protein extraction was performed as described in a previous article [12].

### Liquid chromatography and tandem mass spectrometry

We used a Q-Exactive HF (Thermo Scientific) connected to a Dionex Ultimate NCR-3500RS liquid chromatography (LC) system to analyze the samples, as previously described (Additional files) [11].

### Data analysis

The data were analyzed in the same manner as our previous article [11]. In short, we used Progenesis with default settings for raw data analysis, Proteome Discoverer for protein identification (using the SwissProt human database) and Perseus software (v1.5.0.0) [http://perseus-framework.org/] for the analyses.

### Histology and immunohistochemistry

An experienced nephropathologist (SL) scored the biopsies in a blinded manner using the Oxford classification scoring system [13]. Immunohistochemistry for periostin (Sigma HPA012306, 1:50) was performed after heat-induced antigen retrieval. The tissue glass slides were scanned with ScanScope® XT (Aperio) at × 40. The slides were viewed in ImageScope 12. Periostin expression in glomeruli was quantified by image analysis with the algorithm Positive Pixel Count V9 (Aperio/Leica). Each glomerulus was manually selected and analyzed for pixel intensity; pixels with intensity between 0 and 150 were defined as positive. Number of positive pixels was divided by total number of pixels per glomerulus, thus yielding a proportion of positive pixels per glomeruli. For each patient sample, mean proportion of positive pixels was calculated and these were compared between groups.

### Statistics

The relative differences in protein abundance are given as fold change. Statistical analysis of proteins abundance between groups was performed with Student’s t-test on log transformed intensity data. For other analyses, mean ± standard deviation (SD) is given. Standard two-sided t-tests were used, and *p*-values of < 0.05 were considered statistically significant.

## Results

### Patient characteristics

The clinical characteristics of patients with IgAN (*n* = 25) and of controls (*n* = 15) are shown in Table 1. As compared to controls, IgAN patients were more often male, had more proteinuria and tended to have higher systolic blood pressure. IgAN patients with progressive and non-progressive disease course did not differ in these clinical characteristics. Three of the patients with IgAN had received steroid treatment after diagnosis with at least 20 mg prednisolone daily for at least 1 month (one non-progressor and 2 progressors). Use of angiotensin inhibition treatment has not been reliably registered in the registry.

**Table 1:** Patient’s characteristics

	Controls	IgAN total	IgAN without progression	IgAN with progression
N	15	25	16	9
Year of diagnosis	2000 ± 7.7	1997 ± 4.4	1996 ± 3.4	1998 ± 5.7
Proportion female	53.3%	20%	12.5%	33.3%
Age (years)	32.0 ± 11.9	31.3 ± 14	31.4 ± 13.4	31.2 ± 15.8
Serum creatinine (mmol/l)	79.3 ± 20.9	96.6 ± 23.6 *	91.5 ± 21.5	105.8 ± 25.6*
Estimated glomerular filtration rate ª (ml/min/1.73m^2^)	113.13 ± 18.5	106.16 ± 27.43	114.19 ± 25.3	91.89 ± 26.3*
Systolic blood pressure (mmHg)	118.6 ± 14.6	130.2 ± 19.2	127.2 ± 14.3	135.4 ± 25.8
Diastolic blood pressure (mmHg)	77.6 ± 8.4	78.8 ± 11.9	78.9 ± 11.6	78.8 ± 13.1
Urinary protein (grams/24 h)	0.16 ± 0.17	1.7 ± 1.03 *	1.76 ± 1 *	2.0 ± 2.0 *
Body weight (kg)	74.7 ± 11.9	75.8 ± 9.9	76.1 ± 8.0	75.4 ± 12.5
No of years of follow-up	12.3 ± 7.7		16.3 ± 3.4	
No of years from biopsy to ESRD				5.8 ± 2.5
Percentage with M-score of 1^b^	Not applicable	36%	31.3%	44.4%
Percentage with E-score of 1^b^	Not applicable	32%	31.3%	33.3%
Percentage with S-score of 1^b^	Not applicable	60%	50%	77.8%
Percentage with T-score of 1 or 2^b^	Not applicable	16%	0%	44.4% **
Percentage with C-score of 1^b^	Not applicable	4%	0%	11.1%

*p < 0.05 as compared to control

**p < 0.05 IgAN with progression as compared to IgAN without progression

ª Estimated by CKD-EPI equation

^b^ According to Oxford Classification

### Overall proteome analysis

In total, 3274 different proteins were identified in the analyses, of which 2018 were identified with two or more unique peptides and were included in the quantitative analyses. To detect potential outlier samples, we performed a multi-correlation analysis (Pearson correlation) where each sample-sample correlation was visualized by hierarchical clustering (Additional file 1: Figure S1). The correlation analysis showed high correlation between samples (0.77–0.97) indicating reliable sample processing, microdissection and proteomics and that samples were comparable.

### Extracellular matrix proteome

**Fig. 1:**
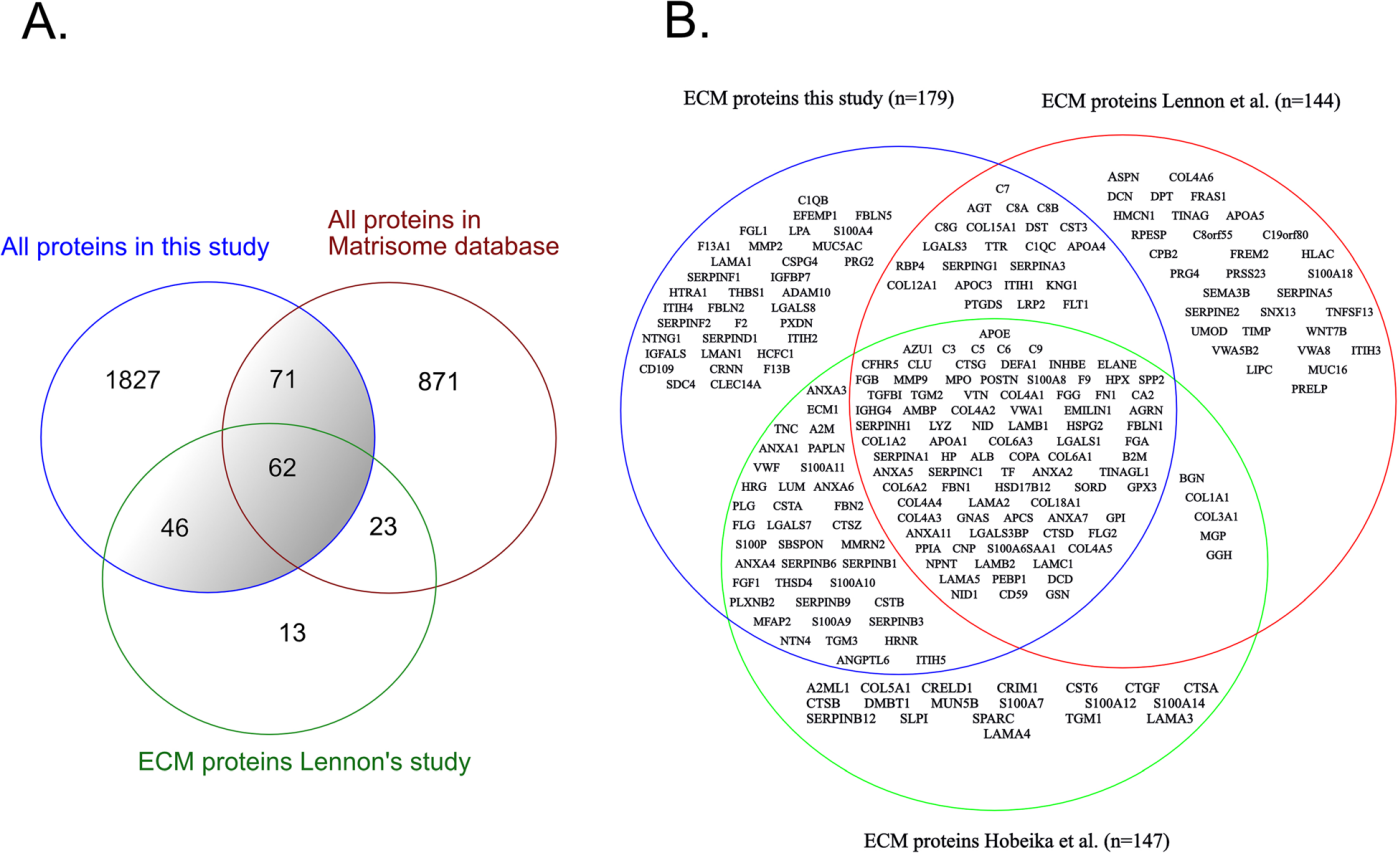
**a** We combined our protein list of quantified proteins with the protein list from the Matrisome project database (*n* = 1027) and the list from Lennon et al. (*n* = 144 proteins). **b**. Comparison of our list of ECM proteins with the protein list from Lennon et al. [7] and Hobeika et al. [8]

Proteins were defined as related to the extracellular matrix if the protein were 1) found in the Matrisome project database (10, 1027 proteins, extracted 15.05.17), or 2) identified in the glomerular ECM enrichment study by Lennon et al. (144 proteins) [7]. This yielded a list of 179 ECM proteins (Fig. 1a, Additional file 2: Table S1). Proteins were grouped using the same approach as Lennon et al. [7].

Of the 179 proteins, 108 had been grouped by Lennon et al. (20 as glomerular basement membrane (GBM) proteins; 14 as other structural ECM proteins and 74 as ECM associated proteins) [7]. The remaining 71 proteins were classified in the Matrisome dataset, either as core proteins (22 proteins) or as Matrisome - associated (49 proteins) and we further classified this proteins using gene ontology annotations into GBM proteins (*n* = 5), structural ECM (*n* = 17) and ECM associated proteins (*n* = 49). Of the 179 proteins, 123 proteins overlapped with the proteins identified by Hobeika et al. who included proteins described by Lennon as well as proteins from their study of microdissected glomeruli that had been described in the Matrisome database (Fig. 1b).

### Basement membrane proteins

We were able to quantify 25 basement membrane proteins (Additional file 3: Table S2). In addition to 20 proteins previously identified by Lennon et al. and Hobeika et al. [7, 8], our dataset contained 5 additional proteins (laminin subunit alpha-1, netrin-4, multimerin-2, papilin and tenascin). Of the basement membrane proteins, 10 were significantly more abundant in IgAN as compared to controls and four of these had a fold change of at least 1.5 (collagen alpha-1 (XV) chain, tenascin, collagen alpha-1 (IV) chain and fibronectin) (Table 2). In the comparison between progressive IgAN as compared to non-progressive IgAN, there were 3 significantly different proteins, 1 was more abundant (von willebrand factor A domain-containing protein 1), and 2 less abundant (agrin and laminin subunit beta-2). By analyzing staining patterns in the human protein atlas [https://www.proteinatlas.org] we found that most of the proteins previously described as basement membrane proteins also showed positive staining in the glomerular mesangium (Table 2).

**Table 2:** Basement membrane proteins identified in our study sorted by fold change between IgAN and control patients. The linear and mesangial matrix staining patterns were assessed by visual inspection of staining in humanproteinatlas.org

Protein Name	Gene Name	Uniprot ID	IgAN total vs control		IgAN progr vs IgA non-progr		Linear GMB staining	Mesangial Matrix staining
			Fold change	*P*-value	Fold change	*P*-value		
Collagen alpha-1(XV) chain	COL15A1	P39059	2.39	0.03	2.30	0.10	Yes	Yes
Tenascin	TNC	P24821	1.85	0.0001	1.16	0.51	Yes	Yes
Collagen alpha-1(IV) chain	COL4A1	P02462	1.54	0.000003	1.10	0.88	Yes	Yes
Fibronectin	FN1	P02751	1.50	0.00004	1.17	0.12	Yes	Yes
Collagen alpha-2(IV) chain	COL5A2	P08572	1.38	0.0003	0.99	0.76	Not detected	Not detected
von Willebrand factor A domain-containing protein 1	VWA1	Q6PCB0	1.36	0.01	1.41	0.01	Pending tissue analysis	
Nidogen-2	NID2	Q14112	1.33	0.0004	1.13	0.62	Yes	Yes
Laminin subunit beta-1	LAMB1	P07942	1.33	0.003	1.05	0.52	Yes	Yes
Basement membrane-specific heparan sulfate proteoglycan core protein	HSPG2	P98160	1.33	0.002	1.08	0.97	Yes	Yes
Fibulin-1	FBLN1	P23142	1.32	0.01	1.35	0.20	Yes	Yes

Only proteins significantly changed between IgAN and controls are shown. Full protein list is shown in Additional file 3: Table S2

### Structural ECM proteins

**Fig. 2:**
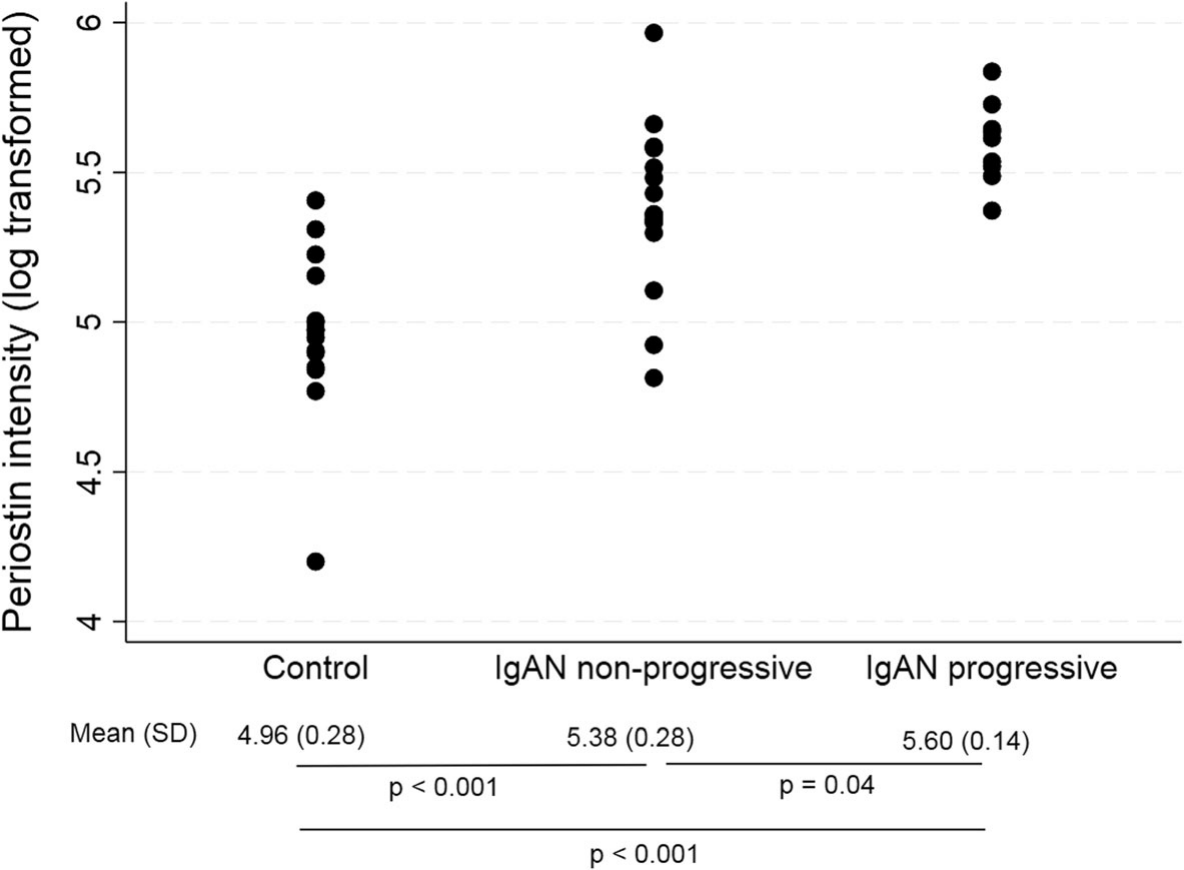
Scatter plot illustrating label-free quantification intensities for Periostin between groups. Mean (standard deviation) and *p*-values are given

We were able to quantify 31 structural ECM proteins (Additional file 4: Table S3). Of these, 14 were previous identified by Lennon et al. and 13 by Hobeika et al. Seventeen proteins were unique to this study. Of the 31 proteins, 11 proteins were significantly different between IgAN and control (Table 3), of which 10 proteins were more abundant and 1 less abundant. Between progressive IgAN and non-progressive IgAN there were 2 significantly more abundant proteins: periostin and fibrinogen-like protein 1. Periostin was the only protein that was significantly more abundant both in IgAN as compared to control as well as in progressive IgAN as compared to non-progressive IgAN, periostin abundance for each patient is shown in Fig. 2.

**Table 3:** Structural ECM proteins identified in our study sorted by fold change between IgAN and control patients

Protein name	Gene Name	Uniprot ID	IgAN total vs control		IgA progr vs IgA non-progr	
			Fold change	*P*-value	Fold change	*P*-value
Periostin	POSTN	Q15063	3.28	0.000001	1.79	0.04
EGF-containing fibulin-like extracellular matrix protein 1	EFEMP1	Q12805	1.89	0.002	1.08	0.98
Fibrinogen beta chain	FGB	P02675	1.87	0.001	1.10	0.71
Vitronectin	VTN	P04004	1.87	0.00001	1.16	0.18
Transforming growth factor-beta-induced protein ig-h3	TGFBI	Q15582	1.80	0.001	1.13	0.65
Extracellular matrix protein 1	ECM1	Q16610	1.78	0.0003	1.30	0.70
Fibulin-5	FBLN5	Q9UBX5	1.76	0.002	0.70	0.85
Netrin-G1	NTNG1	Q9Y2I2	−1.17	0.03	1.19	0.80
Fibrinogen-like protein 1	FGL1	Q08830	1.60	0.04	1.69	0.045
Fibrinogen gamma chain	FGG	P02679	1.53	0.005	0.93	0.61
EMILIN-1	EMILIN1	Q9Y6C2	1.34	0.005	1.06	0.47

Only proteins significantly changed between IgAN and controls are shown. Full protein list is shown in Additional file 4: Table S3

### ECM associated proteins

We quantified totally 123 ECM associated proteins, 74 had been also found in the Lennon et al. study and 80 in the Hobeika et al. study. Thirty-four proteins had not been previously described by Lennon et al. or Hobeika et al. Of these 123 ECM associated proteins, 11 were complement associated proteins and were not investigated further in the present study as they have been discussed separately in a previous paper of the same patients [11]. Of the remaining 112 ECM associated proteins, 32 proteins were significantly different between IgAN and control, 21 were more abundant and 11 less abundant (Table 4). By performing a literature search on these proteins we found that most of them were related to inflammation and immune response (eg azurocidin, myeloperoxidase, neutrophil elastase, cathepsin G, annexin A1, Protein S100-A9 etc), epithelial - mesenchymal transformation (Protein-glutamine gamma-glutamyltransferase 2, Protein S100-A4) and collagen synthesis (serpin H1, MMP2 and MMP9). Between progressive IgAN and non-progressive IgAN there were four significantly different proteins, three were more abundant (clusterin, apolipoprotein E, apolipoprotein A IV,) and one less abundant (carbonic anhydrase 2).

**Table 4:** Significant ECM- associated proteins between IgAN vs Control proteins

Protein name	Gene Name	Uniprot ID	IgAN total vs control		IgAN progr vs IgAN non-progr	
			Fold change	*P*-value	Fold change	*P*-value
Azurocidin	AZU1	P20160	3.31	0.005	1.61	0.11
Secreted phosphoprotein 24	SPP2	Q13103	2.84	0.02	1.56	0.40
Myeloperoxidase	MPO	P05164	2.42	0.0003	1.22	0.08
Neutrophil elastase	ELANE	P08246	2.27	0.006	1.04	0.24
Matrix metalloproteinase-9	MMP9	P14780	2.05	0.0002	1.08	0.12
Neutrophil defensin 1	DEFA1	P59665	1.94	0.0003	1.24	0.27
Protein S100-A8	S100A8	P05109	1.93	0.04	0.94	0.44
Clusterin	CLU	P10909	1.92	0.0001	1.68	0.0004
Protein S100-A4	S100A4	P26447	1.87	0.04	1.67	0.07
Cathepsin G	CTSG	P26447	1.80	0.002	1.56	0.16
Annexin A3	ANXA3	P08311	1.77	0.047	0.97	0.65
Apolipoprotein E	APOE	P12429	1.73	0.00001	1.71	0.03
Protein-glutamine gamma-glutamyltransferase 2	TGM2	P02649	1.67	0.001	1.11	0.54
Protein S100-A9	S100A9	P21980	1.57	0.01	1.04	0.21
72 kDa type IV collagenase (Matrix metalloproteinase-2)	MMP2	P06702	1.48	0.02	1.48	0.46
Annexin A1	ANXA1	P08253	1.45	0.01	1.12	0.82
Protein AMBP	AMBP	P04083	1.42	0.0002	1.32	0.48
CD59 glycoprotein	CD59	P02760	1.39	0.01	1.26	0.14
Serpin H1	SERPINH1	P13987	1.33	0.01	1.16	0.52
Protein S100-A11	S100A11	P50454	1.31	0.01	1.13	0.06
Apolipoprotein A-IV	APOA4	P31949	1.30	0.03	1.41	0.01
Alpha-1-antichymotrypsin	SERPINA3	P06727	1.18	0.02	0.93	0.90
Beta-2-microglobulin	B2M	P01011	1.17	0.047	1.08	0.59
Peptidyl-prolyl cis-trans isomerase A	PPIA	P61769	−1.08	0.04	1.01	0.72
Annexin A2	ANXA2	P62937	−1.14	0.04	1.02	0.87
Phosphatidylethanolamine-binding protein 1	PEBP1	P07355	−1.30	0.003	0.87	0.88
Serpin B9	SERPINB9	P30086	−1.31	0.02	1.06	0.55
Carbonic anhydrase 2	CA2	P50453	−1.38	0.001	0.76	0.02
Galectin-3-binding protein	LGALS3BP	P00918	−1.46	0.01	0.85	0.59
Hornerin	HRNR	Q08380	−1.68	0.04	0.83	0.43
Syndecan-4	SDC4	Q86YZ3	−1.79	0.002	0.88	0.41
Inter-alpha-trypsin inhibitor heavy chain H5	ITIH5	P31431	−1.96	0.0002	1.21	1.00

### Glomerular morphology and periostin staining

**Fig. 3:**
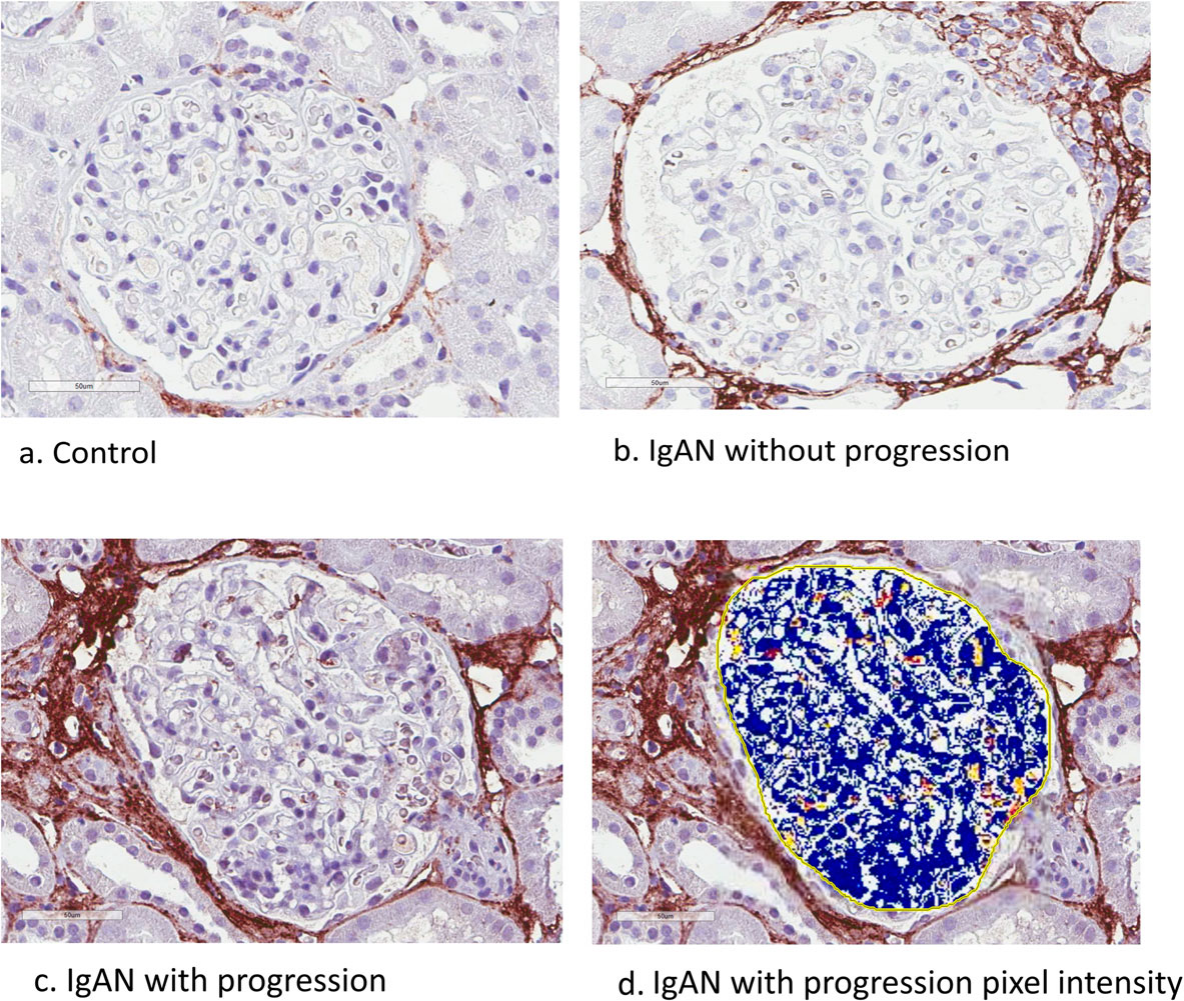
**a**-**d** Periostin staining in the three patient groups **a**) Control patient with negative staining **b**) Non-progressive IgAN patient and **c**) Progressive IgAN patient with more positive staining. There is week periostin staining for both IgAN without and with progression. **d**) Example of Aperio pixel analysis of glomeruli in 3 **c**), orange and yellow indicate positive pixels and blue indicating negative pixels

According to the Oxford classification, 36% of IgAN patients were classified as M1, 32% as E1, 60% as S1, 16% as T1 or T2 and 4% as C1. No patient was classified as C2. As expected, glomeruli from IgAN patients showed more mesangial cell proliferation and matrix expansion as compared to controls. Importantly, Oxford classification of glomerular findings did not differ between non-progressive and progressive IgAN patients, but progressive IgAN patients had more often T1/T2 as compared to non-progressive IgAN patients (0% Vs 44.4%). Immunohistochemistry (IHC) for periostin was performed for 24 samples and examples of staining in the three groups are shown in Figs. 3 a-c, Fig. 3d illustrates pixel analysis. By image analysis, a higher proportion of pixels were positive for periostin in the glomeruli of IgAN as compared with controls (*p*-value = 0.003). There was no significant difference in positivity between progressive and non-progressive IgAN. Interestingly, there was significant periglomerular positivity.

### Could ECM protein abundance separate IgAN patients from control patients?

**Fig. 4:**
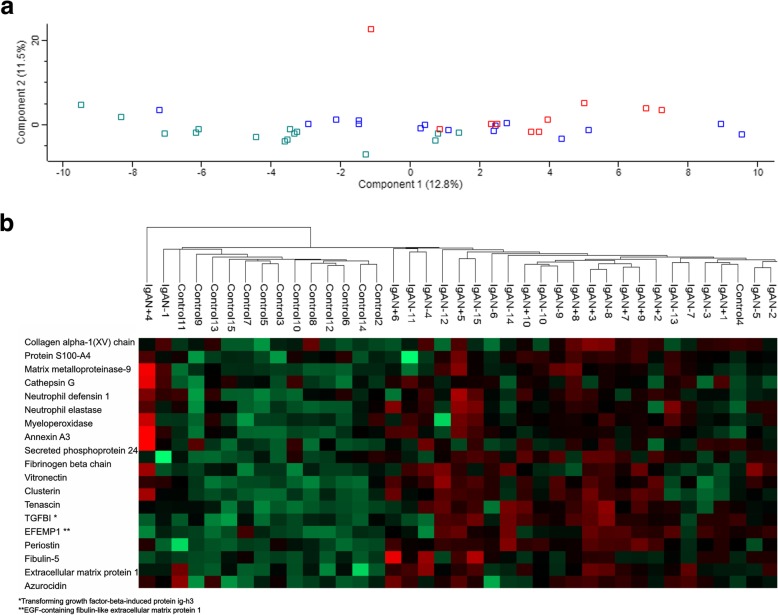
**a** Principal component analysis plot of extracellular matrix proteins. Green represents control patients; blue represents IgAN without progression and red IgAN patients with progression. **b** Unsupervised hierarchical clustering map of the 20 most differently abundant glomerular proteins in patients with IgAN as compared to controls. Each vertical bar represents a patient/control and each horizontal bar represents a protein. Red indicates more abundant proteins while green less abundant proteins in IgAN vs controls. IgA + indicate progressive IgAN patients, IgA – indicate non progressors

As shown in Fig. 4a, principal component plot of the described glomerular ECM proteins showed some separation of control and IgAN patients, but there was significant overlap. Principal component analysis of the 20 most significantly changed proteins did however improve separation (data not shown), this would also be expected but might argue for more direct implications of specific ECM proteins in IgAN. This is further shown in the unsupervised hierarchical clustering map showing relative abundance of these proteins in the individual patients and separation of IgAN and control patients (Fig. 4b).

### Glomerular ECM protein interaction network

**Fig. 5:**
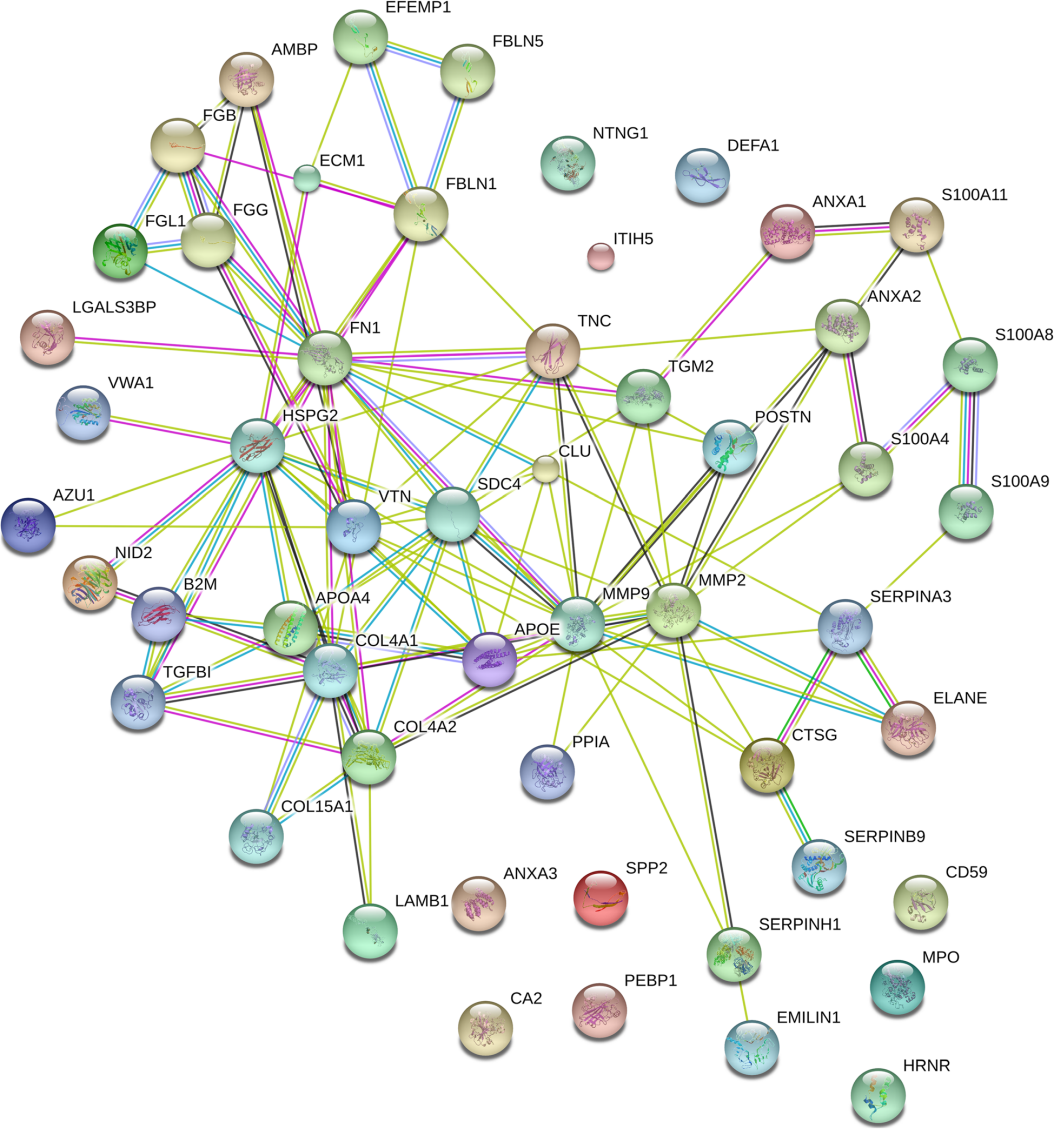
Glomerular ECM protein interaction network. Proteins significantly changed in IgAN glomeruli as compared to control glomeruli were included in the analysis using the STRING database with medium confidence score (0.4). Lines of different color represent types of evidence used in predicting associations. Red line: fusion evidence; green line: neighborhood evidence; blue line: co-occurrence evidence; purple line: experimental evidence; yellow line: text mining evidence; light blue line: database evidence; black line: co-expression evidence. Figure is exported from the string website

All significantly changed ECM proteins identified in the analysis between IgAN and control glomeruli were included in a protein interaction network model using the Search Tool for the Retrieval of Interacting Genes (STRING v10) database with medium confidence score 0.4 (Fig. 5). It was clear that the three groups of proteins that we have used for categorization in the present study interacted strongly. Interestingly, several of the most significantly changed proteins interacted with each other. Periostin interacted for example with fibronectin, MMP2, MMP9, annexin A2 and protein-glutamine gamma-glutamyltransferase 2. Furthermore, collagen alpha-1 (IV) interacted with other collagens, vitronectin as well as TGF-beta induced protein and basement membrane-specific heparan sulfate proteoglycan core protein.

## Discussion

In the present study we have investigated the glomerular extracellular matrix (ECM) proteome in IgAN and quantitatively compared this to the proteome of glomeruli from patients with normal findings on kidney biopsy. We made several interesting findings. First, a high number of ECM associated proteins showed altered abundance in IgAN as compared to controls, several of these were related to inflammation, immune response and fibrosis development. Second, several structural ECM proteins had increased abundance in IgAN compared with controls and the protein with the strongest difference in abundance was periostin. Periostin was also more abundant in patients with progressive IgAN as compared to patients with non-progressive IgAN. Third, basement membrane proteins were increased in IgAN. Overall, our study suggests that glomerular ECM changes in IgAN have strong similarities to changes seen in fibrosis development in general.

In IgAN mesangial IgA deposition and formation of immune complexes leads to mesangial cell proliferation, the release of proinflammatory mediators by mesangial cells and matrix expansion [14]. In our study, most of the ECM associated proteins which had higher abundance in IgAN compared to controls were proteins involved/associated with the immune response and inflammation (eg. azurocidin, myeloperoxidase, neutrophil elastase, cathepsin G, annexin A1, protein S100-A9 etc). Most of these proteins have been described as present in polymorphonuclear leukocytes [15–17], and some may also act as a chemoattractants and activators of monocytes and macrophages [18]. To our knowledge the roles of these proteins have not been described in IgAN, but may mark underlying mechanisms of glomerular damage. Inflammation is for example known to be a propagator of fibrosis development in general [19].

Several structural ECM proteins, such as vitronectin, extracellular matrix protein 1, fibulin-5 and fibrinogen were significantly more abundant in IgAN than in controls. We had not microdissected glomeruli with more than minimal sclerosis and our findings thus most likely illustrate the proteomic changes in mesangial expansion or early glomerular sclerosis. Several proteins that have been shown to be involved in fibrosis development were shown to be more abundant in IgAN compared with controls, such as periostin [20], serpin H1 [21], MMP2 [22] and MMP9 [22, 23]. Periostin was also significantly more abundant in IgAN patients who progressed to ESRD as compared to IgAN patients who did not progress. In the kidney, periostin has been implicated in progression of hypertensive nephropathy [24] and it was increased in glomeruli of patients with progressive proteinuric disease [25]. A recently published study showed that periostin is induced by proinflammatory factors, mainly NFκB in a model of chronic renal disease, and that inhibition of periostin can be used as a therapeutic strategy to slow down renal disease progression [26]. We are not sure why the findings of increased abundance of periostin in progressive IgAN vs non-progressive IgAN from the proteomic analysis were not seen using immunohistochemistry analyses. We believe that the most likely explanation is that mass spectrometry quantification is more precise than quantification by immunohistochemistry.

Important proteins in matrix metabolism, MMP-9 and MMP-2 were also significantly more abundant in IgAN patients compared with controls. MMP-9 and MMP-2 are the most abundant intrarenal metalloproteinases [27] and although it was initially thought that they were mainly implicated in collagen degradation, MMP-2 has been shown to have a pro-inflammatory effect by acting on mesangial cells [28]. Both MMP-2 [29] and MMP-9 [30] are involved in the renal tubular cell epithelial–mesenchymal transition (EMT) and through that promoting fibrosis. Future studies should investigate the roles of these pathways in progressive glomerular sclerosis in IgAN.

In a recent study by Liu et al., genes highly expressed in mesangial cells discriminated better IgAN patients from control patients than genes highly expressed in podocytes and the study thus argued for a strong involvement of mesangial cells in IgAN [31]. Few studies have investigated the proteomic composition of mesangial matrix in IgAN [32], and to our knowledge no previous studies have used modern proteomic approaches. As discussed above, the proteomic changes of the glomerular ECM in our study have strong similarities with those of fibrosis development in general. Mesangial matrix expansion has by many been regarded as a step towards glomerular sclerosis, as was suggested in a review paper by Fogo in 1999 [33], but there seem to be a paucity in data on the underlying mechanisms of this process. Our data and the Liu paper indicate important mechanisms of this process that should be analyzed further.

Several proteins classically described as basement membrane proteins were more abundant in IgAN compared to controls, for example collagen alpha-1 (IV) chain, fibronectin, laminin subunit beta-1, nidogen 1, etc. We could not find studies of thickened GBM in IgAN and previous studies reported rather thinning of GMB in patients with IgAN [34, 35] Using immunostaining Masuda et al. [35] showed reduced α5 (IV) collagen and increased α2 collagen as well as structural changes of α5 (IV) collagen in patients with IgAN. They reported thinning, irregular thickening, small gaps and double contour of GBM examined by *transmission electron microscopy* [35]. By visually inspecting glomerular staining for our proteins in the Human Protein Atlas we did however observe that although these proteins showed clear linear staining of the basement membrane, they also showed positive mesangial staining. We thereby believe that our findings represent changes in the mesangial matrix rather than in the glomerular basement membrane.

The most important strengths of the present study are the large number of quantified proteins from microdissected glomerular tissue. It is also a strength that we included IgAN patients with moderate risk of progressive disease (based on classical prognostic factors), a cohort highly relevant for the clinical nephrologists and that we could separate progressive from non-progressive patients. From a clinical perspective, the number of patients might seem low, but as compared to other proteomic studies this is not the case and we would argue that the similar clinical characteristics of the patients outweigh this limitation. As control group, we used patients with normal kidney biopsy (indication of biopsy was hematuria, proteinuria or reduced eGFR). An extra control group of patients with similar eGFR and another glomerulonephritis would have added more information regarding the particularly proteomic changes that occur in IgAN versus chronic kidney disease in general. It would however have been difficult to know which particular disease to choose for such a comparison as patients with hypertensive nephropathy, FSGS, or lupus nephritis, will also differ from control in several aspects and would have their own proteomic changes. In our opinion, patients with normal kidney biopsy was a good option as control group. As IgAN is an chronic kidney disease we believe that there are many similarities with CKD in general.

Although our control group was defined with GFR over 60 ml/min/1.73m^2^, it is important to mention that only one patient had GFR under 90 ml/min/1.73m^2^ and although the IgAN group was defined as GFR over 45 ml/min/1.73 m ^2^, only one patient had GFR under 60 ml/min/1.73 m ^2^.

The most common indication for biopsy was hematuria (11 patients) or proteinuria (2 patients). This patients did not have known hypertension, diabetes or malignancy and the kidney biopsies were described as normal. It is important to keep in mind that although almost 200 extracellular matrix proteins could be relatively quantified, the exact localization and role of these proteins could not be described. It is for example possible that some of the proteins are deposited intracellularly or have altered levels of activity as compared to normal, we do however not believe this to be the case for the majority of proteins.

## Conclusion

In conclusion our study describes widespread proteome changes of the extracellular matrix in IgAN and implicates several proteins that may be important for development of glomerular damage in IgAN. Most significant proteins were related to inflammation and fibrosis in general and periostin seem to be the most interesting protein as abundance of this protein also could predict progressive IgAN. More studies of the mechanisms linking the described proteins with mesangial expansion and glomerular damage are warranted.

## Supplementary information

**Additional file 1: Figure S1.** Hierarchical clustering show high correlation between samples (0.77 – 0.97) indicating reliable sample processing, microdissection and proteomics.

**Additional file 2:Table S1.** ECM proteins identified in our study (179) arranged by highest change fold between IgAN and control.

**Additional file 3: Table S2.** GBM proteins identified in our study arranged by highest change fold between IgAN and control.

**Additional file 4: Table S3.** Structural ECM proteins identified in our study arranged by highest change fold between IgAN and control.

## Data Availability

The datasets used and/or analyzed during the current study are available from the corresponding author on reasonable request.

## Abbreviations

CKD-EPI: The chronic kidney disease -Epidemiology Collaboration
ECM: Extracellular matrix
EMT: Epithelial–mesenchymal transition
ESRD: End stage renal disease
FFPE: Formalin-fixed paraffin-embedded biopsy
GFR: Glomerular filtration rate
GMB: Glomerular basement membrane
IgAN: IgA nephropathy
IHC: Immunohistochemistry
LC: Liquid chromatography
SD: Standard deviation
