# Supplementary figures and images for: Characterization of glomerular extracellular matrix in IgA nephropathy by proteomic analysis of laser-captured microdissected glomeruli
Source: BMC Nephrol. 2019 Nov 14;20:410. doi: 10.1186/s12882-019-1598-1 (PMC6854890; doi:10.1186/s12882-019-1598-1)

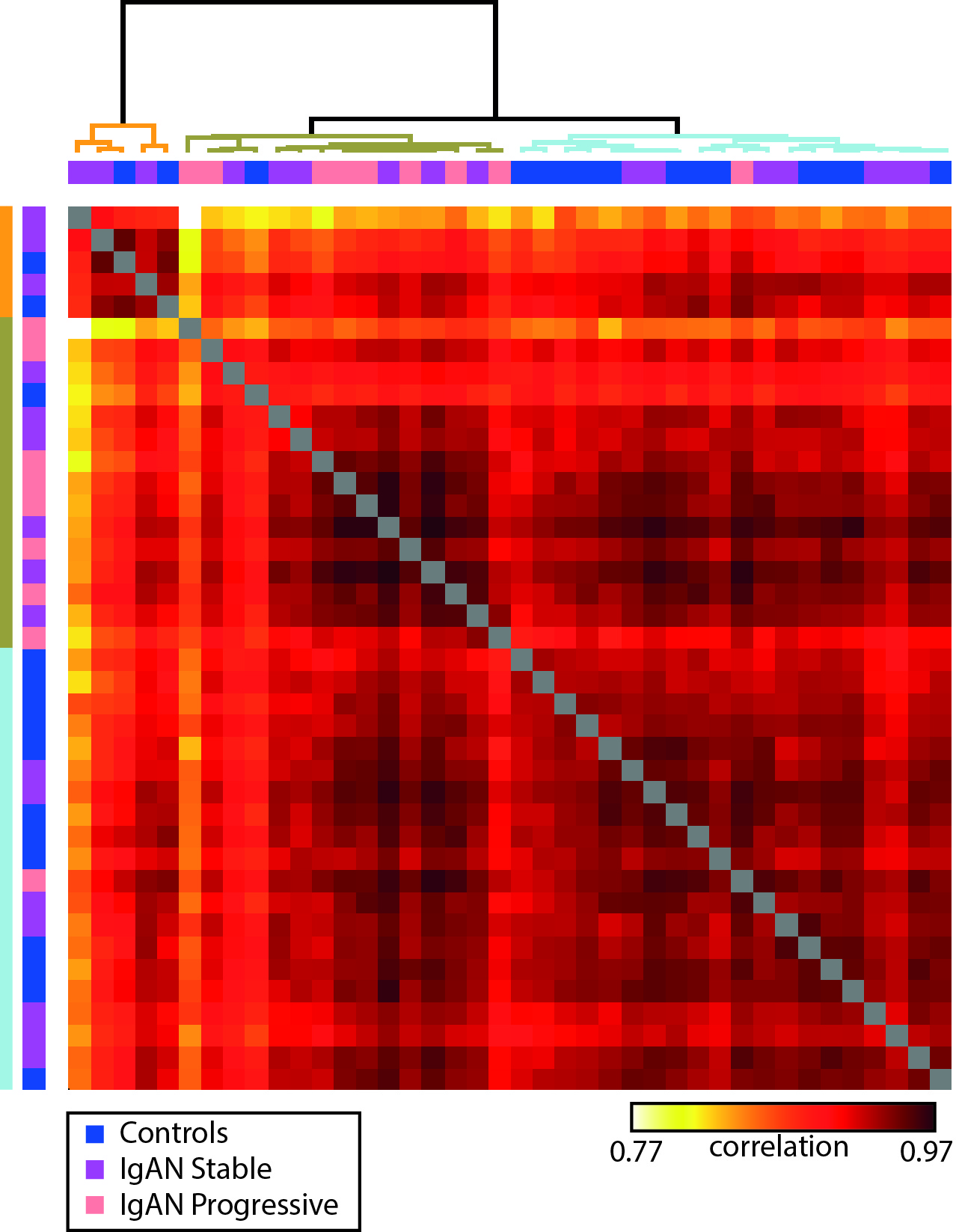

Supplement: Supplementary file 1 — Additional file 1: Figure S1. Hierarchical clustering show high correlation between samples (0.77 – 0.97) indicating reliable sample processing, microdissection and proteomics. [file 12882_2019_1598_MOESM1_ESM.jpg]
