# Supplementary material for: Characterization of glomerular extracellular matrix in IgA nephropathy by proteomic analysis of laser-captured microdissected glomeruli
Source: BMC Nephrol. 2019 Nov 14;20:410. doi: 10.1186/s12882-019-1598-1 (PMC6854890; doi:10.1186/s12882-019-1598-1)
Supplement: Supplementary file 2 — Additional file 2:Table S1. ECM proteins identified in our study (179) arranged by highest change fold between IgAN and control. [file 12882_2019_1598_MOESM2_ESM.docx]

Supplemental Table S1. ECM proteins identified in our study (179) arranged by highest change fold between IgAN and control

|  | Gene Name | Uniprot ID | IgAN total vs control | | IgAN progr vs IgAN non-progr | |
| --- | --- | --- | --- | --- | --- | --- |
|  |  |  | Fold change | P-value | Fold change | P-value |
| Complement component C7 | C7 | P10643 | 5.77 | 0.00000004 | 2.65 | 0.001 |
| Complement component C9 | C9 | P02748 | 5.67 | 0.0000001 | 1.65 | 0.03 |
| Complement component C8 | C8A | P07357 | 3.55 | 0.000001 | 1.97 | 0.001 |
| Azurocidin | AZU1 | P20160 | 3.31 | 0.005 | 1.61 | 0.11 |
| Periostin | POSTN | Q15063 | 3.28 | 0.000001 | 1.79 | 0.04 |
| Complement factor H-related protein 5 | CFHR5 | Q9BXR6 | 3.23 | 0.000002 | 1.79 | 0.01 |
| Complement C5 | C5 | P01031 | 3.22 | 0.0000001 | 1.93 | 0.01 |
| Complement component C8 beta chain | C8B | P07358 | 3.1 | 0.000002 | 1.97 | 0.01 |
| Secreted phosphoprotein | SPP2 | Q13103 | 2.84 | 0.02 | 1.56 | 0.4 |
| Complement component C6 | C6 | P13671 | 2.81 | 0.000004 | 2.95 | 0.0003 |
| Myeloperoxidase | MPO | P05164 | 2.42 | 0.0003 | 1.22 | 0.08 |
| Collagen alpha-1(XV) chain | COL15A1 | P39059 | 2.39 | 0.03 | 2.3 | 0.1 |
| Neutrophil elastase | ELANE | P08246 | 2.27 | 0.006 | 1.04 | 0.24 |
| Complement C3 | C3 | P01024 | 2.13 | 0.000001 | 1.46 | 0.01 |
| Matrix metalloproteinase-9 | MMP9 | P14780 | 2.05 | 0.0002 | 1.08 | 0.12 |
| Neutrophil defensin 1 | DEFA1 | P59665 | 1.94 | 0.0003 | 1.24 | 0.27 |
| Protein S100-A8 | S100A8 | P05109 | 1.93 | 0.04 | 0.94 | 0.44 |
| Clusterin | CLU | P10909 | 1.92 | 0.0001 | 1.68 | 0.0004 |
| Apolipoprotein(a) | LPA | P08519 | 1.91 | 0.11 | 2.58 | 0.004 |
| EGF-containing fibulin-like extracellular matrix protein 1 | EFEMP1 | Q12805 | 1.89 | 0.002 | 1.08 | 0.98 |
| Fibrinogen beta chain | FGB | P02675 | 1.87 | 0.001 | 1.1 | 0.71 |
| Vitronectin | VTN | P04004 | 1.87 | 0.00001 | 1.16 | 0.18 |
| Protein S100-A4 | S100A4 | P26447 | 1.87 | 0.04 | 1.67 | 0.07 |
| Tenascin | TNC | P24821 | 1.85 | 0.0001 | 1.16 | 0.51 |
| Cathepsin G | CTSG | P08311 | 1.8 | 0.002 | 1.56 | 0.16 |
| Transforming growth factor-beta-induced protein ig-h3 | TGFBI | Q15582 | 1.8 | 0.001 | 1.13 | 0.65 |
| Extracellular matrix protein 1 | ECM1 | Q16610 | 1.78 | 0.0003 | 1.3 | 0.7 |
| Annexin A3 | ANXA3 | P12429 | 1.77 | 0.05 | 0.97 | 0.65 |
| Fibulin-5 | FBLN5 | Q9UBX5 | 1.76 | 0.002 | 0.7 | 0.85 |
| Low-density lipoprotein receptor-related protein 2 | LRP2 | P98164 | 1.76 | 0.74 | 0.67 | 0.03 |
| Apolipoprotein E | APOE | P02649 | 1.73 | 0.00001 | 1.71 | 0.03 |
| Protein-glutamine gamma-glutamyltransferase 2 | TGM2 | P21980 | 1.67 | 0.001 | 1.11 | 0.54 |
| Protein S100-P | S100P | P25815 | 1.64 | 0.77 | 1.4 | 0.95 |
| Fibrinogen-like protein 1 | FGL1 | Q08830 | 1.6 | 0.04 | 1.69 | 0.05 |
| Dystonin | DST | Q03001 | 1.59 | 0.46 | 0.98 | 0.67 |
| Protein S100-A9 | S100A9 | P06702 | 1.57 | 0.01 | 1.04 | 0.21 |
| Complement component C8 gamma chain | C8G | P07360 | 1.54 | 0.00002 | 1.72 | 0.0002 |
| Collagen alpha-1(IV) chain | COL4A1 | P02462 | 1.54 | 0.000003 | 1.1 | 0.88 |
| Fibrinogen gamma chain | FGG | P02679 | 1.53 | 0.005 | 0.93 | 0.61 |
| Coagulation factor XIII A chain | F13A1 | P00488 | 1.52 | 0.64 | 1.02 | 0.5 |
| Complement C1q subcomponent subunit B | C1QB | P02746 | 1.5 | 0.001 | 1.18 | 0.57 |
| Fibronectin | FN1 | P02751 | 1.5 | 0.00004 | 1.17 | 0.12 |
| Lysozyme C | LYZ | P61626 | 1.49 | 0.1 | 0.7 | 0.73 |
| Cystatin-C | CST3 | P01034 | 1.48 | 0.07 | 0.86 | 0.76 |
| 72 kDa type IV collagenase | MMP2 | P08253 | 1.48 | 0.02 | 1.48 | 0.46 |
| Ig gamma-4 chain C region | IGHG4 | P01861 | 1.47 | 0.86 | 0.96 | 0.72 |
| Annexin A1 | ANXA1 | P04083 | 1.45 | 0.01 | 1.12 | 0.82 |
| Protein AMBP | AMBP | P02760 | 1.42 | 0.0002 | 1.32 | 0.48 |
| Galectin-3 | LGALS3 | P17931 | 1.4 | 0.27 | 1.26 | 0.83 |
| CD59 glycoprotein | CD59 | P13987 | 1.39 | 0.01 | 1.26 | 0.14 |
| Collagen alpha-2(IV) chain | COL4A2 | P08572 | 1.38 | 0.0003 | 0.99 | 0.76 |
| von Willebrand factor A domain-containing protein 1 | VWA1 | Q6PCB0 | 1.36 | 0.01 | 1.41 | 0.01 |
| Papilin | PAPLN | O95428 | 1.34 | 0.14 | 0.96 | 0.72 |
| EMILIN-1 | EMILIN1 | Q9Y6C2 | 1.34 | 0.005 | 1.06 | 0.47 |
| Serpin H1 | SERPINH1 | P50454 | 1.33 | 0.01 | 1.16 | 0.52 |
| Nidogen-2 | NID2 | Q14112 | 1.33 | 0.0004 | 1.13 | 0.62 |
| Laminin subunit beta-1 | LAMB1 | P07942 | 1.33 | 0.003 | 1.05 | 0.52 |
| Basement membrane-specific heparan sulfate proteoglycan core protein | HSPG2 | P98160 | 1.33 | 0.002 | 1.08 | 0.97 |
| von Willebrand factor | VWF | P04275 | 1.32 | 0.2 | 1.57 | 0.23 |
| Fibulin-1 | FBLN1 | P23142 | 1.32 | 0.01 | 1.35 | 0.2 |
| Protein S100-A11 | S100A11 | P31949 | 1.31 | 0.01 | 1.13 | 0.06 |
| Transthyretin | TTR | P02766 | 1.3 | 0.27 | 0.63 | 0.28 |
| Complement C1q subcomponent subunit C | C1QC | P02747 | 1.3 | 0.09 | 1.54 | 0.04 |
| Apolipoprotein A-IV | APOA4 | P06727 | 1.3 | 0.03 | 1.41 | 0.01 |
| Mucin-5AC | MUC5AC | P98088 | 1.29 | 0.13 | 1.35 | 0.35 |
| Laminin subunit alpha-1 | LAMA1 | P25391 | 1.27 | 0.09 | 1.14 | 0.39 |
| Chondroitin sulfate proteoglycan 4 | CSPG4 | Q6UVK1 | 1.25 | 0.31 | 1.26 | 0.97 |
| Retinol-binding protein 4 | RBP4 | P02753 | 1.24 | 0.23 | 1.22 | 0.66 |
| Bone marrow proteoglycan | PRG2 | P13727 | 1.2 | 0.78 | 0.78 | 0.98 |
| Pigment epithelium-derived factor | SERPINF1 | P36955 | 1.19 | 0.08 | 0.93 | 0.86 |
| Insulin-like growth factor-binding protein 7 | IGFBP7 | Q16270 | 1.19 | 0.21 | 0.97 | 0.25 |
| Plasma protease C1 inhibitor | SERPING1 | P05155 | 1.18 | 0.95 | 1.51 | 0.26 |
| Alpha-1-antichymotrypsin | SERPINA3 | P01011 | 1.18 | 0.02 | 0.93 | 0.9 |
| C-type lectin domain family 14 member A | CLEC14A | Q86T13 | 1.17 | 0.7 | 1.33 | 0.95 |
| Beta-2-microglobulin | B2M | P61769 | 1.17 | 0.05 | 1.08 | 0.59 |
| Histidine-rich glycoprotein | HRG | P04196 | 1.16 | 0.6 | 0.76 | 0.14 |
| Collagen alpha-3(VI) chain | COL6A3 | P12111 | 1.16 | 0.38 | 0.89 | 0.4 |
| Galectin-1 | LGALS1 | P09382 | 1.15 | 0.18 | 1.13 | 0.15 |
| Lumican | LUM | P51884 | 1.15 | 0.57 | 1 | 0.55 |
| Fibrinogen alpha chain | FGA | P02671 | 1.14 | 0.06 | 1.42 | 0.45 |
| Alpha-1-antitrypsin | SERPINA1 | P01009 | 1.14 | 0.26 | 0.83 | 0.86 |
| Coagulation factor IX | F9 | P00740 | 1.14 | 0.17 | 1.33 | 0.32 |
| Coatomer subunit alpha | COPA | P53621 | 1.13 | 0.07 | 1.26 | 0.1 |
| Serine protease HTRA1 | HTRA1 | Q92743 | 1.13 | 0.21 | 1.14 | 0.29 |
| Collagen alpha-1(VI) chain | COL6A1 | P12109 | 1.12 | 0.34 | 1 | 0.57 |
| Annexin A6 | ANXA6 | P08133 | 1.11 | 0.51 | 1.14 | 0.15 |
| Thrombospondin-1 | THBS1 | P07996 | 1.1 | 0.44 | 1.07 | 0.6 |
| Plasminogen | PLG | P00747 | 1.1 | 0.29 | 1.23 | 0.07 |
| Haptoglobin | HP | P00738 | 1.09 | 0.58 | 1.07 | 0.71 |
| Cystatin-A | CSTA | P01040 | 1.09 | 0.98 | 0.88 | 0.52 |
| Fibrillin-2 | FBN2 | P35556 | 1.08 | 0.54 | 1.04 | 0.96 |
| Alpha-2-macroglobulin | A2M | P01023 | 1.07 | 0.31 | 1.35 | 0.05 |
| Annexin A5 | ANXA5 | P08758 | 1.07 | 0.17 | 0.79 | 0.1 |
| Antithrombin-III | SERPINC1 | P01008 | 1.06 | 0.07 | 0.98 | 0.84 |
| Tubulointerstitial nephritis antigen-like | TINAGL1 | Q9GZM7 | 1.06 | 0.5 | 1.12 | 0.33 |
| Collagen alpha-1(XII) chain | COL12A1 | Q99715 | 1.05 | 0.92 | 1.08 | 0.73 |
| Collagen alpha-2(VI) chain | COL6A2 | P12110 | 1.05 | 0.73 | 1.06 | 0.67 |
| Fibrillin-1 | FBN1 | P35555 | 1.05 | 0.46 | 1.38 | 0.1 |
| Disintegrin and metalloproteinase domain-containing protein 10 | ADAM10 | O14672 | 1.05 | 0.6 | 1.16 | 0.84 |
| Galectin-7 | LGALS7 | P47929 | 1.04 | 0.67 | 0.98 | 0.39 |
| Protein S100-A10 | S100A10 | P60903 | 1.04 | 0.96 | 1.38 | 0.03 |
| Very-long-chain 3-oxoacyl-CoA reductase | HSD17B12 | Q53GQ0 | 1.03 | 0.38 | 1.37 | 0.23 |
| Cathepsin Z | CTSZ | Q9UBR2 | 1.03 | 0.46 | 1.42 | 0.35 |
| Sorbitol dehydrogenase | SORD | Q00796 | 1.03 | 0.55 | 0.91 | 0.3 |
| Somatomedin-B and thrombospondin type-1 domain-containing protein | SBSPON | Q8IVN8 | 1.03 | 0.76 | 0.93 | 0.72 |
| Collagen alpha-4(IV) chain | COL4A4 | P53420 | 1.01 | 0.38 | 0.66 | 0.41 |
| Multimerin-2 | MMRN2 | Q9H8L6 | 1.01 | 0.87 | 1.18 | 0.24 |
| Inter-alpha-trypsin inhibitor heavy chain H1 | ITIH1 | P19827 | -1 | 0.86 | 1.15 | 0.87 |
| Hemopexin | HPX | P02790 | -1 | 0.82 | 1.32 | 0.8 |
| Laminin subunit alpha-2 | LAMA2 | P24043 | -1 | 0.98 | 0.81 | 0.21 |
| Inter-alpha-trypsin inhibitor heavy chain H4 | ITIH4 | Q14624 | -1.01 | 0.23 | 1.26 | 0.34 |
| Collagen alpha-1(XVIII) chain | COL18A1 | P39060 | -1.02 | 0.18 | 1.03 | 0.95 |
| Serotransferrin | TF | P02787 | -1.02 | 0.59 | 0.79 | 0.12 |
| Collagen alpha-3(IV) chain | COL4A3 | Q01955 | -1.03 | 1 | 0.66 | 0.12 |
| Fibulin-2 | FBLN2 | P98095 | -1.05 | 0.93 | 1.81 | 0.31 |
| Serum amyloid P-component | APCS | P02743 | -1.06 | 0.68 | 1.04 | 0.64 |
| Annexin A7 | ANXA7 | P20073 | -1.06 | 0.5 | 0.9 | 0.13 |
| Glucose-6-phosphate isomerase | GPI | P06744 | -1.06 | 0.31 | 0.94 | 0.95 |
| Nidogen-1 | NID1 | P14543 | -1.06 | 0.31 | 0.83 | 0.15 |
| Galectin-8 | LGALS8 | O00214 | -1.07 | 0.67 | 1.24 | 0.19 |
| Annexin A4 | ANXA4 | P09525 | -1.07 | 0.75 | 0.88 | 0.07 |
| Alpha-2-antiplasmin | SERPINF2 | P08697 | -1.07 | 0.27 | 0.91 | 0.4 |
| Annexin A11 | ANXA11 | P50995 | -1.08 | 0.3 | 0.95 | 0.43 |
| Serpin B6 | SERPINB6 | P35237 | -1.08 | 0.1 | 0.79 | 0.15 |
| Cathepsin D | CTSD | P07339 | -1.08 | 0.75 | 0.98 | 0.92 |
| Prothrombin | F2 | P00734 | -1.08 | 0.73 | 1.06 | 0.97 |
| Peroxidasin homolog | PXDN | Q92626 | -1.08 | 0.68 | 1.31 | 0.21 |
| Peptidyl-prolyl cis-trans isomerase A | PPIA | P62937 | -1.08 | 0.04 | 1.01 | 0.72 |
| Filaggrin-2 | FLG2 | Q5D862 | -1.09 | 0.89 | 0.71 | 0.4 |
| Gelsolin | GSN | P06396 | -1.09 | 0.1 | 1.12 | 0.9 |
| Leukocyte elastase inhibitor | SERPINB1 | P30740 | -1.09 | 0.24 | 0.92 | 0.39 |
| 2'.3'-cyclic-nucleotide 3'-phosphodiesterase | CNP | P09543 | -1.1 | 0.61 | 1.44 | 0.86 |
| Protein S100-A6 | S100A6 | P06703 | -1.1 | 0.55 | 0.83 | 0.32 |
| Glutathione peroxidase 3 | GPX3 | P22352 | -1.11 | 0.96 | 1.01 | 0.4 |
| Serum albumin | ALB | P02768 | -1.11 | 0.26 | 0.79 | 0.27 |
| Serum amyloid A-1 protein | SAA1 | P0DJI8 | -1.11 | 0.45 | 0.8 | 0.8 |
| Fibroblast growth factor 1 | FGF1 | P05230 | -1.11 | 0.3 | 0.77 | 0.08 |
| Collagen alpha-5(IV) chain | COL4A5 | P29400 | -1.12 | 0.92 | 0.7 | 0.19 |
| Thrombospondin type-1 domain-containing protein 4 | THSD4 | Q6ZMP0 | -1.13 | 0.46 | 1.35 | 0.1 |
| Guanine nucleotide-binding protein G(s) subunit alpha isoforms XLas | GNAS | Q5JWF2 | -1.13 | 0.18 | 0.82 | 0.03 |
| Nephronectin | NPNT | Q6UXI9 | -1.14 | 0.7 | 1.03 | 0.87 |
| Annexin A2 | ANXA2 | P07355 | -1.14 | 0.04 | 1.02 | 0.87 |
| Angiotensinogen | AGT | P01019 | -1.15 | 0.41 | 2.26 | 0.03 |
| Kininogen-1 | KNG1 | P01042 | -1.15 | 0.76 | 0.99 | 0.67 |
| Agrin | AGRN | O00468 | -1.16 | 0.16 | 0.79 | 0.02 |
| Laminin subunit beta-2 | LAMB2 | P55268 | -1.16 | 0.1 | 0.72 | 0.02 |
| Laminin subunit gamma-1 | LAMC1 | P11047 | -1.17 | 0.14 | 0.74 | 0.12 |
| Netrin-G1 | NTNG1 | Q9Y2I2 | -1.17 | 0.03 | 1.19 | 0.8 |
| Prostaglandin-H2 D-isomerase | PTGDS | P41222 | -1.19 | 0.22 | 0.97 | 0.83 |
| Heparin cofactor 2 | SERPIND1 | P05546 | -1.21 | 0.62 | 1.3 | 0.14 |
| Inter-alpha-trypsin inhibitor heavy chain H2 | ITIH2 | P19823 | -1.22 | 0.27 | 1.11 | 0.22 |
| Insulin-like growth factor-binding protein complex acid labile subunit | IGFALS | P35858 | -1.24 | 0.58 | 1.2 | 0.5 |
| Laminin subunit alpha-5 | LAMA5 | O15230 | -1.25 | 0.09 | 0.79 | 0.05 |
| Plexin-B2 | PLXNB2 | O15031 | -1.27 | 0.14 | 1.01 | 0.34 |
| Protein ERGIC-53 | LMAN1 | P49257 | -1.27 | 0.16 | 1 | 0.61 |
| Filaggrin | FLG | P20930 | -1.29 | 0.4 | 1.52 | 0.08 |
| Phosphatidylethanolamine-binding protein 1 | PEBP1 | P30086 | -1.3 | 0.003 | 0.87 | 0.88 |
| Apolipoprotein C-III | APOC3 | P02656 | -1.31 | 0.11 | 1.42 | 0.04 |
| Serpin B9 | SERPINB9 | P50453 | -1.31 | 0.02 | 1.06 | 0.55 |
| Inhibin beta E chain | INHBE | P58166 | -1.36 | 0.45 | 1.31 | 0.78 |
| Carbonic anhydrase 2 | CA2 | P00918 | -1.38 | 0.001 | 0.76 | 0.02 |
| Host cell factor 1 | HCFC1 | P51610 | -1.4 | 0.06 | 0.83 | 0.73 |
| Cystatin-B | CSTB | P04080 | -1.42 | 0.09 | 1.06 | 0.05 |
| Vascular endothelial growth factor receptor 1 | FLT1 | P17948 | -1.42 | 0.06 | 0.52 | 0.004 |
| Microfibrillar-associated protein 2 | MFAP2 | P55001 | -1.43 | 0.95 | 1.45 | 0.37 |
| Serpin B3 | SERPINB3 | P29508 | -1.45 | 0.76 | 0.93 | 0.8 |
| Netrin-4 | NTN4 | Q9HB63 | -1.46 | 0.56 | 0.71 | 0.4 |
| Galectin-3-binding protein | LGALS3BP | Q08380 | -1.46 | 0.01 | 0.85 | 0.59 |
| Apolipoprotein A-I | APOA1 | P02647 | -1.47 | 0.08 | 1.55 | 0.03 |
| Dermcidin | DCD | P81605 | -1.48 | 0.1 | 0.93 | 0.59 |
| Protein-glutamine gamma-glutamyltransferase E | TGM3 | Q08188 | -1.52 | 0.77 | 0.63 | 0.13 |
| CD109 antigen | CD109 | Q6YHK3 | -1.52 | 0.2 | 1.26 | 0.29 |
| Cornulin | CRNN | Q9UBG3 | -1.53 | 0.63 | 1.28 | 0.24 |
| Coagulation factor XIII B chain | F13B | P05160 | -1.62 | 0.94 | 1.04 | 0.28 |
| Hornerin | HRNR | Q86YZ3 | -1.68 | 0.04 | 0.83 | 0.43 |
| Collagen alpha-2(I) chain | COL1A2 | P08123 | -1.73 | 0.49 | 1.53 | 0.83 |
| Angiopoietin-related protein 6 | ANGPTL6 | Q8NI99 | -1.73 | 0.37 | 1.25 | 0.88 |
| Syndecan-4 | SDC4 | P31431 | -1.79 | 0.002 | 0.88 | 0.41 |
| Inter-alpha-trypsin inhibitor heavy chain H5 | ITIH5 | Q86UX2 | -1.96 | 0.0002 | 1.21 | 1 |
