# Supplementary material for: Characterization of glomerular extracellular matrix in IgA nephropathy by proteomic analysis of laser-captured microdissected glomeruli
Source: BMC Nephrol. 2019 Nov 14;20:410. doi: 10.1186/s12882-019-1598-1 (PMC6854890; doi:10.1186/s12882-019-1598-1)
Supplement: Supplementary file 3 — Additional file 3: Table S2. GBM proteins identified in our study arranged by highest change fold between IgAN and control. [file 12882_2019_1598_MOESM3_ESM.docx]

Supplemental table S2. GBM proteins identified in our study arranged by highest change fold between IgAN and control

|  | IgA total vs control | | Progressive IgA vs non progressive | |
| --- | --- | --- | --- | --- |
|  | Fold change | P-value | Fold change | P-value |
| Collagen alpha-1(XV) chain | 2.39 | 0.03 | 2.30 | 0.10 |
| Tenascin | 1.85 | 0.0001 | 1.16 | 0.51 |
| Collagen alpha-1(IV) chain | 1.54 | 0.000003 | 1.10 | 0.88 |
| Fibronectin | 1.50 | 0.00004 | 1.17 | 0.12 |
| Collagen alpha-2(IV) chain | 1.38 | 0.0003 | 0.99 | 0.76 |
| von Willebrand factor A domain-containing protein 1 | 1.36 | 0.01 | 1.41 | 0.01 |
| Nidogen-2 | 1.33 | 0.00 | 1.13 | 0.62 |
| Laminin subunit beta-1 | 1.33 | 0.00 | 1.05 | 0.52 |
| Basement membrane-specific heparan sulfate proteoglycan core protein | 1.33 | 0.00 | 1.08 | 0.97 |
| Fibulin-1 | 1.32 | 0.01 | 1.35 | 0.20 |
| Papilin | 1.34 | 0.14 | 0.96 | 0.72 |
| Laminin subunit alpha-1 | 1.27 | 0.09 | 1.14 | 0.39 |
| Fibrillin-1 | 1.05 | 0.46 | 1.38 | 0.10 |
| Collagen alpha-4(IV) chain | 1.01 | 0.38 | 0.66 | 0.41 |
| Multimerin-2 | 1.01 | 0.87 | 1.18 | 0.24 |
| Laminin subunit alpha-2 | -1.00 | 0.98 | 0.81 | 0.21 |
| Collagen alpha-1(XVIII) chain | -1.02 | 0.18 | 1.03 | 0.95 |
| Collagen alpha-3(IV) chain | -1.03 | 1.00 | 0.66 | 0.12 |
| Nidogen-1 | -1.06 | 0.31 | 0.83 | 0.15 |
| Collagen alpha-5(IV) chain | -1.12 | 0.92 | 0.70 | 0.19 |
| Agrin | -1.16 | 0.16 | 0.79 | 0.02 |
| Laminin subunit beta-2 | -1.16 | 0.10 | 0.72 | 0.02 |
| Laminin subunit gamma-1 | -1.17 | 0.14 | 0.74 | 0.12 |
| Laminin subunit alpha-5 | -1.25 | 0.09 | 0.79 | 0.05 |
| Netrin-4 | -1.46 | 0.56 | 0.71 | 0.40 |
