# Supplementary material for: Characterization of glomerular extracellular matrix in IgA nephropathy by proteomic analysis of laser-captured microdissected glomeruli
Source: BMC Nephrol. 2019 Nov 14;20:410. doi: 10.1186/s12882-019-1598-1 (PMC6854890; doi:10.1186/s12882-019-1598-1)
Supplement: Supplementary file 4 — Additional file 4: Table S3. Structural ECM proteins identified in our study arranged by highest change fold between IgAN and control. [file 12882_2019_1598_MOESM4_ESM.docx]

Supplemental Table S3. Structural ECM proteins identified in our study arranged by highest change fold between IgAN and control

|  | IgAN total vs control | | IgAN progr vs IgAN non-progr | |
| --- | --- | --- | --- | --- |
|  | Fold change | P-value | Fold change | P-value |
| Periostin | 3.28 | 0.000001 | 1.79 | 0.04 |
| EGF-containing fibulin-like extracellular matrix protein 1 | 1.89 | 0.002 | 1.08 | 0.98 |
| Fibrinogen beta chain | 1.87 | 0.001 | 1.10 | 0.71 |
| Vitronectin | 1.87 | 0.00001 | 1.16 | 0.18 |
| Transforming growth factor-beta-induced protein ig-h3 | 1.80 | 0.001 | 1.13 | 0.65 |
| Extracellular matrix protein 1 | 1.78 | 0.0003 | 1.30 | 0.70 |
| Fibulin-5 | 1.76 | 0.002 | 0.70 | 0.85 |
| Fibrinogen-like protein 1 | 1.60 | 0.04 | 1.69 | 0.045 |
| Fibrinogen gamma chain | 1.53 | 0.005 | 0.93 | 0.61 |
| EMILIN-1 | 1.34 | 0.00 | 1.06 | 0.47 |
| von Willebrand factor | 1.32 | 0.20 | 1.57 | 0.23 |
| Bone marrow proteoglycan | 1.20 | 0.78 | 0.78 | 0.98 |
| Insulin-like growth factor-binding protein 7 | 1.19 | 0.21 | 0.97 | 0.25 |
| Collagen alpha-3(VI) chain | 1.16 | 0.38 | 0.89 | 0.40 |
| Lumican | 1.15 | 0.57 | 1.00 | 0.55 |
| Fibrinogen alpha chain | 1.14 | 0.06 | 1.42 | 0.45 |
| Collagen alpha-1(VI) chain | 1.12 | 0.34 | 1.00 | 0.57 |
| Thrombospondin-1 | 1.10 | 0.44 | 1.07 | 0.60 |
| Fibrillin-2 | 1.08 | 0.54 | 1.04 | 0.96 |
| Tubulointerstitial nephritis antigen-like | 1.06 | 0.50 | 1.12 | 0.33 |
| Collagen alpha-1(XII) chain | 1.05 | 0.92 | 1.08 | 0.73 |
| Collagen alpha-2(VI) chain | 1.05 | 0.73 | 1.06 | 0.67 |
| Somatomedin-B and thrombospondin type-1 domain-containing protein | 1.03 | 0.76 | 0.93 | 0.72 |
| Fibulin-2 | -1.05 | 0.93 | 1.81 | 0.31 |
| Peroxidasin homolog | -1.08 | 0.68 | 1.31 | 0.21 |
| Thrombospondin type-1 domain-containing protein 4 | -1.13 | 0.46 | 1.35 | 0.10 |
| Nephronectin | -1.14 | 0.70 | 1.03 | 0.87 |
| Netrin-G1 | -1.17 | 0.03 | 1.19 | 0.80 |
| Insulin-like growth factor-binding protein complex acid labile subunit | -1.24 | 0.58 | 1.20 | 0.50 |
| Microfibrillar-associated protein 2 | -1.43 | 0.95 | 1.45 | 0.37 |
| Collagen alpha-2(I) chain | -1.73 | 0.49 | 1.53 | 0.83 |
